# Biomaterial Drug Delivery Systems for Prominent Ocular Diseases

**DOI:** 10.3390/pharmaceutics15071959

**Published:** 2023-07-15

**Authors:** Avin Sapowadia, Delaram Ghanbariamin, Libo Zhou, Qifa Zhou, Tannin Schmidt, Ali Tamayol, Yupeng Chen

**Affiliations:** 1 Department of Biomedical Engineering, University of Connecticut, Storrs, CT 06269, USA; avin.sapowadia@uconn.edu (A.S.); delaram@uconn.edu (D.G.); zhoulibo1030@163.com (L.Z.); tschmidt@uchc.edu (T.S.); atamayol@uchc.edu (A.T.); 2 Department of Biomedical Engineering, University of Connecticut Health Center, Farmington, CT 06030, USA; 3 Department of Biomedical Engineering and Ophthalmology, University of Southern California, Los Angeles, CA 90089, USA; qifazhou@usc.edu

**Keywords:** ocular drug delivery, nanoparticles, biomaterials, ocular implants, hydrogels, DNA-inspired janus base nanopieces

## Abstract

Ocular diseases, such as age-related macular degeneration (AMD) and glaucoma, have had a profound impact on millions of patients. In the past couple of decades, these diseases have been treated using conventional techniques but have also presented certain challenges and limitations that affect patient experience and outcomes. To address this, biomaterials have been used for ocular drug delivery, and a wide range of systems have been developed. This review will discuss some of the major classes and examples of biomaterials used for the treatment of prominent ocular diseases, including ocular implants (biodegradable and non-biodegradable), nanocarriers (hydrogels, liposomes, nanomicelles, DNA-inspired nanoparticles, and dendrimers), microneedles, and drug-loaded contact lenses. We will also discuss the advantages of these biomaterials over conventional approaches with support from the results of clinical trials that demonstrate their efficacy.

## 1. Introduction

Ocular diseases are conditions or disorders that negatively affect eye functioning and result in visual impairment, and they are more prevalent in older populations [1]. A study surveying 39 countries indicated that the number of people of all ages that are visually impaired is estimated to be 285 million, where individuals aged 50 and older represent 65% and 82% of the visually impaired and blind, respectively [2]. Systematic reviews evaluating the impact of different ocular diseases on quality of life have consistently shown a reduction in functional status and independence. Decreased emotional well-being and psychosocial functioning have also been found to be associated with ocular diseases, and ophthalmic interventions have shown a positive association with quality of life [3]. These findings support efforts to improve the efficacy of treatments for ocular diseases and access to these treatments.

**Figure 1:**
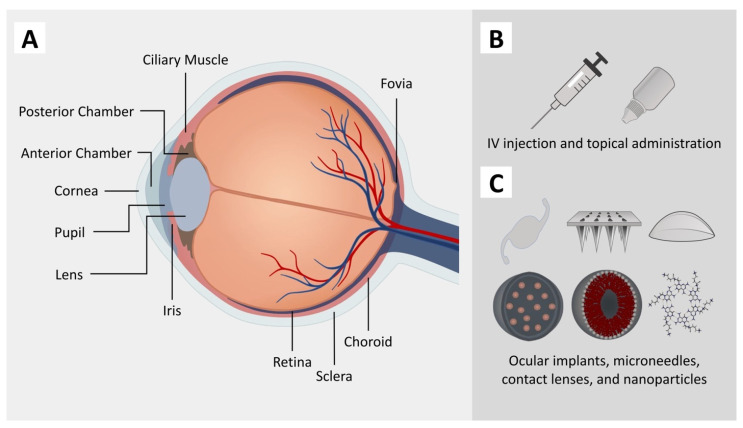
Schematic representation of different sections of the human eye (**A**), conventional drug delivery techniques (**B**), and novel drug delivery techniques (**C**).

The structure of the eye can be divided into the anterior segment, including the cornea, aqueous humor, and conjunctiva, and the posterior segment, including the retina, sclera, and choroid (Figure 1). Ocular diseases are categorized accordingly based on the tissue or section impaired. Glaucoma, as an anterior ocular disease, and age-related macular degeneration (AMD) and diabetic retinopathy, as posterior ocular diseases, are known for being some of the most pervasive causes of visual impairment and blindness globally and directly undermine the quality of life of affected individuals [4]. Many of these diseases share similar risk factors, including age, hypertension, prolonged exposure to sunlight, and nutritional factors such as Vitamin E [5].

While the identified risk factors help examine individuals for the presence of ocular diseases, more efforts have been made to develop possible treatments. Efficient drug delivery through routes such as topical, intravitreal, and periocular administration is considered to be the most common way of treating ocular diseases. The delivery approach in the treatment of ocular diseases differs based on the affected site. Topical administration of therapeutics is the most common route of drug delivery to the anterior segment, and intravitreal and periocular injections are some of the most preferable methods for the treatment of posterior ocular diseases [6,7]. However, treatment options for almost all ocular diseases are limited due to pharmaceutical barriers mostly caused by the anatomy and physiology of the eye [8]. Precorneal factors and ocular barriers, which consist of different mechanisms to protect the eyes, pose challenges to drug delivery to the target site and drug permeation in topical administration methods [9,10]. Drugs are typically either retained at the cornea and/or conjunctiva or need to reach the eye’s internal structures depending on their target sites [11]. When retained at the conjunctiva, drugs are absorbed systemically through the conjunctival blood capillaries and lymphatics, significantly decreasing the ocular bioavailability of the administered drugs. The permeation of a drug through the sclera is also dependent on the drug’s molecular radius and surface charge due to its composition [12,13]. As a result, the cornea serves as the main site for drug delivery for pathologies located in inner eye structures in topical drug administration. The highly organized multilayered corneal structure is the main challenge for drugs crossing the corneal barrier [11]. In other administration routes, such as IV injection, multiple risk factors exist in addition to patient noncompliance and post-treatment monitoring, such as retinal detachment and hemorrhage, despite the higher bioavailability of the drugs [14]. Periocular administration of drugs is also known as a successful approach for treating ocular diseases; however, there are still some drawbacks of using this route that limit efficient drug delivery, such as an increase in intraocular pressure or drug elimination by choroidal circulation [8,15,16].

Thus, it is necessary to design novel targeted drug delivery systems/techniques to overcome these challenges. A wide range of drug delivery systems based on biomaterials have been developed as possible treatments for different ocular diseases with the aim of increasing the ocular bioavailability of drugs and reducing the adverse side effects of these therapeutics. These attempts are mostly focused on increasing drug residency time on the ocular surface, improving the physiochemical properties of therapeutics for higher corneal penetration, and sustaining the delivery of drugs [17]. In this review paper, a few examples of the most common ocular diseases and their conventional treatment methods will be discussed to identify existing challenges and the potential for development. We then highlight some of the major classes of biomaterials developed for ocular drug delivery purposes, along with specific examples, recent improvements, and applications.

## 2. Prominent Ocular Diseases and Conventional Treatment Techniques

Before the advancement in nanodrug delivery carriers, ocular diseases were treated using a variety of systems, though these have proven to be suboptimal [18,19]. The majority of ophthalmic products have been topical formulations due to convenience, including eye drops, eye ointments, and contact lenses [10,12]. While many techniques differed between different ocular diseases, many shared similar limitations, such as low bioavailability, poor drug absorption, and poor medication adherence [20]. For the purposes of this review, the most prominent ocular diseases and their respective treatments will be discussed, and similarities between them will be indicated. 

### 2.1. Age-Related Macular Degeneration

With the increase in life expectancy, age-related diseases frequently occur in many different organ systems, such as the cardiovascular system [21], musculoskeletal system [22,23], and nervous system [24]. Age-related macular degeneration (AMD) is a notable disease in the visual system and can be classified as early or late AMD. Early AMD is associated with pigmentary changes in the macula in individuals aged over fifty, resulting in few visual symptoms, while late AMD is associated with significant visual loss and is further classified into dry and wet AMD [25]. Wet AMD, also known as neovascular AMD, is characterized by the presence of choroidal neovascularization—development of new blood vessels in the choroid layer behind the retina—and associated manifestations, such as retinal pigment epithelial detachment and subretinal hemorrhages [26]. Most research studies and therapies have focused their attention on this type of AMD with the aim of reducing choroidal neovascularization. The advanced form of AMD is referred to as geographic atrophy, which is characterized by the presence of demarcated atrophic lesions of the outer retina [27]. 

Beginning in the 1980s, the Macular Photocoagulation Study Group shared favorable outcomes involving the use of thermal laser photocoagulation in a small proportion of the eye [28]. Photocoagulation of subfoveal choroidal neovascularization was recommended for patients with lesions resulting in a loss in visual acuity (VA) below 20/200. Photodynamic therapy was introduced in the late 1990s. This procedure involved intravenous administration of a pharmacological photosensitizer such as verteporfin. Light was used to induce photochemical oxidation of vascular endothelia, ensuring that no thermal tissue damage occurred [29]. However, this type of therapy was most effective for the classic angiographic subtype of wet AMD, which represents 18% to 24% of choroidal neovascularization lesions [30]. 

One of the more recent treatments is anti-vascular endothelial growth factor (VEGF) therapy. VEGF is a key component to the pathogenesis of choroidal neovascularization. VEGF inhibitors are a class of drugs that have been established as the standard of care and are typically given via intravitreal injection [31]. In 2004, pegaptanib (Macugen, Pfizer)—a small oligonucleic acid molecule that binds to the VEGF-165 isoform—became the first Food and Drug Administration (FDA)-approved drug for AMD treatment [32]. Other anti-VEGF drugs were soon developed, increasing the pool of acceptable drugs for AMD treatment. Some examples of anti-angiogenic agents that attempt to block various steps in the pathway of angiogenesis in choroidal neovascularization include bevacizumab (Avastin), ranibizumab (rhuFab V2; Lucentis), anecortave acetate (Retaane), triamcinolone acetonide, squalamine lactate (Evizon), Sirna-027 (small interfering RNA, siRNA), and VEGF-Trap (VEGF-TrapR1R2; soluble decoy receptor) [33]. Similar to Sirna-027, bevasiranib, a novel siRNA-based therapeutic, has been believed to down-regulate VEGF production in the retina and may serve as a possible treatment for wet AMD [34]. Due to the abundance of anti-VEGF inhibitors, some studies have attempted to assess and differentiate the compounds by comparing binding kinetics and relative biological activity. One study shows that VEGF-Trap binds to VEGF-A, the most studied member of the VEGF family, with higher affinity than ranibizumab or bevacizumab [35]. However, a more recent study demonstrates in a rabbit model that the half-life of VEGF-Trap (3.63 days) is still shorter than that of bevacizumab (6.99 days) [36].

Intravitreal injections can deliver sufficient amounts of anti-VEGF drugs, but frequent injections are required since drug molecules are rapidly cleared from the vitreous. This may increase the risk of retinal detachment, hemorrhage, and endophthalmitis [37]. Additionally, the pharmacokinetics of such drugs have been closely investigated, revealing the role of charge and molecular weight differences on vitreous distribution and elimination [38].

### 2.2. Glaucoma

Glaucoma affects nearly 80 million people worldwide and is known as the leading cause of blindness [39]. This ocular disease can be mainly classified as either open-angle glaucoma or angle-closure glaucoma, where both are characterized by the degeneration of retinal ganglion cells and resulting changes in the optic nerve head. While other factors may also play a role, the degeneration of retinal ganglion cells is related to the level of intraocular pressure [40]. The goal for glaucoma treatment is slowing the progression of the disease, which centers on reducing intraocular pressure. 

Since 1856, many surgical procedures have been developed to treat acute and chronic glaucoma. Relief of pupillary block, external filtration, internal filtration, and ciliodestruction are techniques that have been used for surgery [41]. More recently, surgeries have focused on reducing intraocular pressure by increasing outflow and decreasing the inflow of the aqueous humor [41]. If surgery is not necessary, the first line of treatments typically includes topical selective and nonselective beta-blockers or prostaglandin analogs. Alpha-agonists and topical carbonic anhydrase can also be used [42]. If a certain therapy is not effective, combination therapy is recommended. Limitations of the techniques and therapies mentioned exist. Commercially available eyedrops show low compliance (due to experiencing difficulties in administering eye drops and ocular discomfort, especially in the elderly population), short therapeutic time, and low bioavailability due to precorneal factors, while glaucoma surgeries, as an invasive treatment method, always have a risk of failure due to enhanced fibrosis [43,44]. 

### 2.3. Diabetic Retinopathy and Diabetic Macular Edema

Characterized by hyperglycemia, basement membrane thickening, pericyte loss, and preretinal neovascularization, diabetic retinopathy can lead to blindness through hemorrhage and tractional retinal detachment [45]. Hyperglycemia appears to be the greatest risk factor, which contributes to the change in the balance of regulators throughout the retinal vasculature. Among many mechanisms that have been proposed for how retinal capillary damage occurs, the main mechanisms include increased polyol pathways, activation of protein kinase C, increased non-enzymatic glycation, and the generation of reactive oxygen species [45]. Moreover, diabetic retinopathy caused by hyperglycemia-induced events regulates the synthesis of growth factors, specifically from the VEGF and pigment epithelium-derived factor (PEDF) families. 

In the 1970s, some clinical trials indicated that laser photocoagulation could be an effective therapy for diabetic retinopathy [46,47]. Laser photocoagulation may decrease the risk of vitreous hemorrhage and membrane formation by regressing newly formed blood vessels due to normalizing the partial pressure of oxygen in the peripheral avascular areas of the retina [48]. In many cases, the optimal time for laser treatment is passed, and it is not uniformly successful in halting visual decline. Additionally, if argon laser photocoagulation is used, this may lead to moderate visual loss, a diminished visual field, reduced color vision, and reduced contrast sensitivity [49]. In terms of pharmacotherapy, intravitreal injection of glucocorticoids or VEGF antagonists may be used, but the same problems result from using this form of treatment: the effects are short-lasting, limited to only about four to six weeks [48]. Similar to the treatment for AMD, using VEGF antagonists via intravitreal injections may result in complications such as retinal detachment or may have deleterious effects on the remaining healthy retina.

Diabetic macular edema, characterized by the swelling of the macula due to leaking of blood vessels, is a complication that may result from diabetic retinopathy. Similar to that of diabetic retinopathy, argon laser photocoagulation as a treatment has been shown to reduce the risk of visual loss for patients with clinically significant diabetic macular edema [50]. Additionally, the use of anti-VEGF agents may also be used to target the VEGF-driven processes of neovascularization and blood–retinal barrier dysfunction (Table 1) [51].

## 3. Ocular Implants

Ocular implants have evolved significantly over the past several decades and aim to act as drug reservoirs which can controllably release drugs for a prolonged period. Before the advancement of biodegradable ocular implants, non-biodegradable implants were developed and involved surgical procedures for implantation. Due to certain drawbacks, such as the requirement for a second procedure to remove the implant after depleting the drug load, biodegradable implants were later developed to enhance the patient experience. 

### 3.1. Non-Biodegradable Materials

Non-biodegradable implants are materials that typically do not face any changes in structure during implantation and are not affected by the surrounding environment. Such materials can be transplanted directly into the vitreous body but can cause complications similar to intravitreal injections due to the invasive procedure [54]. Some non-biodegradable implants are introduced to the eye in the form of a matrix (monolithic) system, where the drug of interest is dispersed homogeneously. Other non-biodegradable implants may take the form of a reservoir, where a compact drug core is surrounded by a permeable non-biodegradable membrane [55]. Based on a number of factors, such as the thickness of membranes, the release area, the permeability of the drug through the membrane, and the solubility of the drug, the reservoir-type non-biodegradable implant can be modified to achieve a desired drug release rate [56]. Additionally, non-biodegradable implants can take the form of a pump through the use of microelectromechanical system technology, where the implant can be small enough for ocular use [57].

As discussed earlier, anti-VEGF agents are useful for the treatment of ocular diseases like AMD or diabetic macular edema. Non-biodegradable implants that are devised as intravitreal depots containing anti-VEGF medications can allow for the prolonged diffusion of these medications to the posterior segment of the eye. In 2014, a novel Posterior MicroPump Drug Delivery System (PMP) for patients with diabetic macular edema was developed and tested to report device capabilities for delivering ranibizumab, the anti-VEGF drug, using microelectromechanical system technology [58]. Once the PMP was prefilled with the anti-VEGF drug and implanted into the subconjunctival space, the PMP was wirelessly controlled to deliver a programmed microdose in the vitreous cavity. Benchtop data collected by the researchers show that the PMP device can continue to function reliably for more than 100 programmable injections of intraocular drugs like ranibizumab. The Phase I clinical trial involving patients with diabetic macular edema indicates that the use of PMP for the sustained release of ranibizumab was successful for 90 days without surgical complications before extraction [58]. While researchers have reported favorable outcomes, further studies investigating longer-term safety are still needed. 

While anti-VEGF agents can act directly on VEGF to decrease vascular permeability, corticosteroids can also achieve this while additionally inhibiting inflammatory cytokines and multiple chemokines [59]. In general, corticosteroids are thought to act as inhibitors of phospholipase A_2_ via activation of lipocortins, which results in halting of the release of arachidonic acid—a common precursor for inflammatory mediators [60]. Therefore, corticosteroids can be used for the treatment of diabetic macular edema. Fluocinolone acetonide is one such corticosteroid that has been used as a non-biodegradable, injectable intravitreal implant. The fluocinolone acetonide intravitreal implant containing 0.19 mg of active drug (ILUVIEN)—approved by several countries, including the United States—releases fluocinolone acetonide at an initial rate of 0.25 µg/day (average rate 0.2 µg/day) and lasts for approximately 36 months [60]. In order to evaluate the efficacy of the fluocinolone acetonide intravitreal implant in diabetic macular edema, a pair of Fractional Flow Reserve Versus Angiography for Multivessel Evaluation (FAME) Phase 3 trials were conducted [61,62]. The results from both randomized, sham injection-controlled trials were pooled together for analysis. FAME analysis showed a greater visual acuity improvement in patients with the fluocinolone acetonide intravitreal implant compared to the sham injection. This result was significant as early as three weeks and remained significant after 24 months and 36 months. Other studies and reports have also indicated the efficacy of fluocinolone acetonide intravitreal implants [63,64,65]. 

The Port Drug Delivery (PDS) system with ranibizumab (Roche/Genentech Inc., San Francisco, CA, USA) is a novel reservoir-type drug delivery device that can be surgically implanted into the vitreous cavity to allow for the continuous, effective release of an anti-VEGF drug. This system can be used as a treatment for ocular diseases like neovascular AMD or diabetic retinopathy, eliminating the need for frequent intravitreal injections to deliver an anti-VEGF drug [66]. At 2.6 mm in width and 8.4 mm in length, the non-biodegradable PDS system is a refillable implant composed of polysulfone. One concern during the Phase 2 study was the high incidence of vitreous hemorrhage post surgery. After optimizing the surgical implantation technique, vitreous hemorrhage was reduced to 5.2% (from approximately 50%) in the Phase 3 study [67]. For drug replenishment, the PDS system may not require extraction due to the presence of a self-sealing septum in the implant that remains accessible via the conjunctiva; thus, a needle can be used to replenish the drug [67]. This is an important aspect in the development of these non-biodegradable implants since extraction of the device may cause complications and discomfort for the patient. In October 2021, the United States FDA approved the use of the PDS with ranibizumab for clinical treatment of neovascular AMD [68].

Currently, the iDose^®^ TR intraocular implant is being tested through a Phase 3 clinical trial designed to compare the safety and efficacy of the travoprost-containing implant to a timolol maleate ophthalmic solution (0.5%) for patients with open-angle glaucoma (NCT03519386). The researchers will be investigating two different elution rates of travoprost, which is known to reduce intraocular pressure. Previous Phase 2 clinical trials using the implant have shown reductions in intraocular pressure from baseline of 33% and 32% by the fast-release and slow-release implants, respectively, with no serious adverse events [69]. Table 2 summarizes notable and ongoing clinical trials using the different biomaterials mentioned in this review. 

### 3.2. Biodegradable Materials

Implantable devices that can be broken down into smaller nontoxic fragments, which can subsequently be absorbed or excreted by the body, are known as biodegradable implants. A significant drawback of the use of non-biodegradable implants is the need for extraction—a second surgical procedure. Biodegradable implants were developed to overcome this issue by only requiring one surgical procedure and are often made from polymers such as polycaprolactone (PCL), polylactide (PLA), and poly(lactic-co-glycolic acid) (PLGA) [55,76]. These polymers can be eliminated safely from the human body while limiting the impact on eye health. Additionally, biodegradable implants can take the form of many shapes through the process of compression molding, extrusion, or solution casting, including but not limited to plugs, pellets, discs, and sheets [77]. A “final burst stage” is possible, causing uncontrollable release of the remaining drug within the implant, which typically starts after the biodegradable polymer reaches the critical point of hydrolysis. Thus, rigorous testing is required to ensure proper release of the drug load. Furthermore, control over the degradation rate of biodegradable implants is essential and can be influenced by many factors, such as the percentage of loaded drug, the water solubility of the drug, the surface area of the implant, and the speed of polymer degradation [78]. 

PLGA has been used for many applications, such as bone tissue engineering, due to its biocompatibility, tailored biodegradation rate, and approval for clinical use in humans by the United States FDA [79]. It can easily be made into a variety of shapes for these applications, such as screws, rods, and plates. In regards to ocular diseases, a highly developed PLGA-based intravitreal implant called Ozurdex has been used for the treatment of diabetic macular edema after being approved by the FDA in June 2014 [80]. Ozurdex (DEX Implant 0.7 mg, Ozurdex, Allergan Inc., Dublin, Ireland) releases dexamethasone, a potent corticosteroid with anti-inflammatory activity and the potential to diminish vessel leakage, into the vitreous cavity [81]. The three-year randomized and sham-controlled trial of the use of Ozurdex for patients with diabetic macular edema from 2014 revealed statistically significant and clinically meaningful long-term improvement in vision [80]. In 2016, a published study evaluated the efficacy of Ozurdex in patients with persistent diabetic macular edema. The implant resulted in an improvement in Early Treatment Diabetic Retinopathy Study (ETDRS) outcomes, which assess best-corrected visual acuity (BCVA) at different times post injection (one month, three months, four months, and six months after injection) [82]. When Ozurdex is used for patients with neovascular AMD however, results do not indicate visual benefits. This was tested in pilot studies published in 2016 and 2018, where the implant combined with an anti-VEGF therapy (ranibizumab) resulted in improved retinal fluid resorption but no significant change in BCVA [83,84]. One potential downside of the use of Ozurdex is ocular hypertension (increased intraocular pressure). Ocular hypertension was evaluated in a study in 2017 which collected data from retrospective consecutive case series with Ozurdex implantation recipients. Approximately 26.9% of implantation recipients showed ocular hypertension [85]. While the study may be affected by selection bias, more studies are needed to identify the possibility of glaucoma in these patients. 

In 2002, two identical randomized clinical trials showed that bimatoprost—a prostaglandin analog—can reduce intraocular pressure when administered topically [86]. Researchers have used this information to develop a biodegradable implant for the treatment of glaucoma. Results from a Phase 1/2 pair-eyed controlled clinical trial were published in 2017, indicating that a bimatoprost sustained-release implant (Durysta, Allergan) provided a rapid and sustained decrease in intraocular pressure through six months in open-angle glaucoma patients [87]. In 2020, researchers also shared favorable efficacy and safety profiles over 24 months following implantation [75]. Durysta delivers bimatoprost using a solid polymer platform for drug delivery composed of the aliphatic polyesters poly(D,L-lactide), poly(D,L-lactide-co-glycolide), poly(D,L-lactide) acid end, and polyethylene glycol 3350 [88,89]. The implant was approved by the United States FDA in March 2020 for the treatment of open-angle glaucoma and ocular hypertension [89].

Brimonidine, an alpha-2 adrenergic receptor agonist, has previously been discovered to lower intraocular pressure and provide neuroprotection and has been considered as a monotherapy, adjunctive therapy, and replacement therapy in open-angle glaucoma [90]. More recently, however, an intravitreal implant containing brimonidine in a poly-(D,L-lactide) biodegradable polymer implant was developed and tested for the potential treatment of geographic atrophy, an advanced form of AMD [74]. The randomized, sham-controlled, multicenter (25 clinical sites in seven countries) Phase 2 study reported consistently smaller geographic area growth when using the Brimonidine Drug Delivery System (Brimo DDS) compared to the sham and demonstrated a favorable safety profile [74]. Phase 3 development is supported. 

More recently, Parrag et al. developed a non-polymeric, fully biodegradable intravitreal implant (IBE-814 IVT implant) designed to deliver a low dose of dexamethasone for up to 6 months [91]. Using New Zealand and Dutch Belted rabbits, the researchers were able to achieve low, consistent dose release from the implant onto the vitreous humor, retina, and choroid for up to 12 months with no drug detected at 14 months. A Phase II clinical trial is ongoing to evaluate the safety and efficacy of the implant in patients with diabetic macular edema and is expected to be completed by 2024. 

Biodegradable hydrogels can also be used as implants for ocular drug delivery. The highly porous three-dimensional networks are typically made of hydrophilic polymers crosslinked physically (Van der Waals forces, hydrogen bonding, hydrophobic forces, or ionic bonding) or chemically (covalent bonding) and are able to swell while retaining their structure [92,93]. Hydrogels have high hydrophilicity due to the presence of hydrophilic moieties distributed along the backbone of polymeric chains, such as carboxyl, amino, hydroxyl, and amide groups [94,95]. For the treatment of ocular diseases, hydrogels can be of use due to their high biocompatibility and ability to hold and release therapeutic drugs. They can be classified by their origin (natural, synthetic, or synthetic/natural hybrid polymers) and can also be classified by their durability (durable or degradable), structure (amorphous or semi-crystalline), charge, response to stimuli (temperature, pH, ionic strength, etc.), and composition (homopolymer, copolymer, or semi-interpenetrating network) [94]. The controlled release of therapeutic drugs within hydrogels can be described as follows: an initial burst release phase due to a high surface-to-volume ratio, a diffusion-dominated phase, and a final hydrogel degradation-dominated phase [96]. Hydrogels for ophthalmic applications typically need to be biodegradable, nontoxic, and biocompatible, just like other biomaterials mentioned in this review. They must be able to overcome challenges such as sterilization before application, which can alter the structure of the hydrogel and change its release profile [97]. Based on the classification scheme mentioned above, it is evident that several types of hydrogels can be constructed for drug delivery. 

Clinical trials have been started for a punctal plug delivery device, which is placed in the eye’s tear ducts. Vantipalli et al. evaluated the safety and efficacy of a hydrogel-based insert delivering travoprost—a prostaglandin analog—in a multicenter Phase 3 clinical trial for open-angle glaucoma and ocular hypertension patients in 2020 [98]. The rod-shaped hydrogel was incorporated into the travoprost punctal plug, OTX-TP (Ocular Therapeutix, Inc., Bedford, MA, USA), which was placed in the eye’s tear duct. The resolvable polyethylene glycol (PEG) hydrogel can swell to fill the canalicular space to gradually release travoprost over a 90-day period. Among the 554 patients with open-angle glaucoma or ocular hypertension, OTX-TP-treated patients had greater statistically significant reductions in intraocular pressure relative to the placebo insert at eight of the nine different time points (8 a.m., 10 a.m., and 4 p.m. at 2, 6, and 12 weeks following insertion). While transient mild adverse events were observed in 7% of the OTX-TP-treated patients, they were mostly resolved over time [98]. Based on the results, the clinical trial did not show an exceptional reduction in intraocular pressure, which suggests that more testing is needed. 

More recently, a Phase 1 clinical trial sponsored by PolyActiva Pty Ltd., Victoria, Australia (NCT04060758) began recruiting participants to test a polytriazole-based hydrogel on participants with open-angle glaucoma. The researchers aim to identify a safe and officious dose of latanoprost-free acid using biodegradable hydrogel. Komaromy et al. previously demonstrated reductions in intraocular pressure from hydrogel implants after the sustained release of latanoprost-free acid over 6 months [99].

In situ fabrication of hydrogels has become an interesting approach for the treatment of ocular diseases. In this formulation, some protein-based polymers can self-assemble; motifs such as elastin and silk have been explored in the past, and have been considered for use in the treatment of ocular diseases [100,101]. Fernández-Colino et al. in 2017 evaluated the performance of both elastin-like and silk-like hydrogels on a macroscopic scale by conducting rheological tests [102]. Both hydrogels tested contained timolol maleate as the active ingredient, which reduces intraocular pressure by blocking beta adrenoreceptors in the ciliary body of the eye [103] and is comparable to bimatoprost for the treatment of glaucoma. The hydrogels were able to facilitate drug incorporation at low temperatures and transformed into the gel form at physiological temperature for sustained release when used topically. For topical administration of the hydrogels, in vivo adhesion tests and intraocular pressure measurements performed on New Zealand rabbits showed prolonged retention on the preocular surface and reduced intraocular pressure. While these hydrogels are novel and versatile, further testing and preparation for clinical trials are needed. 

## 4. Injectable Nanocarriers

**Figure 2:**
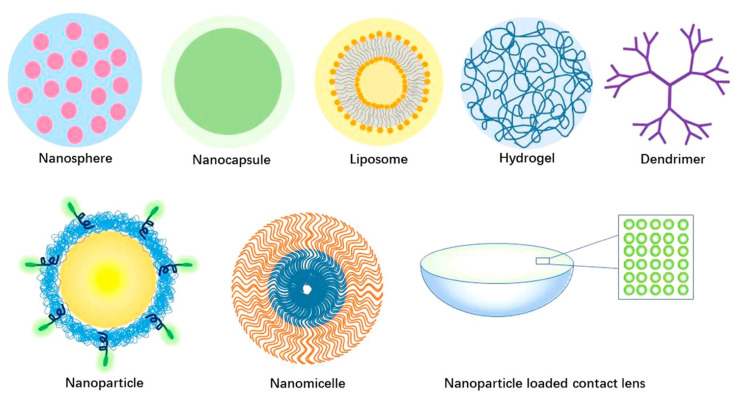
Different nanocarriers for ocular drug delivery. Reproduced from Weng et al. [110] under the Creative Commons Attribution 4.0 International License (CC BY 4.0).

The term nanocarrier is a broad term that is used to describe exceptionally small (10–1000 nm) particles that can transport a therapeutic drug at a specific site and then act as a drug depot, thus enabling continuous drug delivery [104,105]. Due to their small size, nanocarriers offer many advantages. For instance, nanocarriers can travel through the endothelium in inflammatory sites or epithelium and, more notably, can efficiently be drawn into several different cell types for selective drug accumulation at target sites [104,106]. Additionally, the use of nanocarriers in liquid or semisolid formulations has been shown to prolong the residence time of ophthalmic drugs [9]. The surface charge of nanocarriers, measured as zeta potential, also plays a role in cellular uptake and in drug release. Nanocarriers have broad applications, including the encapsulation of anticancer agents to control metastasis, and have gained attention for their ability to modulate various physiological or pathological processes [107,108,109]. Among the many novel injectable nanocarriers that have been developed, including nanospheres, nanocapsules, and nanosuspensions, hydrogels, liposomes, nanomicelles, DNA-inspired materials, and dendrimers are discussed in the context of prominent ocular diseases. Figure 2 shows a schematic of some of the nanocarriers used for ocular drug delivery [110]. 

### 4.1. Hydrogels

Aside from being used as implants, hydrogels can also be made as a nanocarrier formulation to carry an ocular drug for injectable use. These formulations are also known as hydrogel particles due to their dimensions being in the order of nanometers or micrometers.

As mentioned earlier, bevacizumab (Avastin) has been used clinically for the treatment of neovascular AMD due to its anti-VEGF character and has also been used in a hydrogel. Wang et al. synthesized a biocompatible material made of a triblock copolymer of poly(2-ethyl-2-oxazoline)-*b*-poly(ε-caprolactone)-*b*-poly(2-ethyl-2-oxazoline) (PEOz-PCL-PEOz) for the sustained release of bevacizumab [111]. PEOz-PCL-PEOz was synthesized in a three-step process, and its aqueous solution, ECE hydrogel, can encapsulate bevacizumab. Since the hydrogel was thermosensitive—similar to Fernández-Colino et al.’s elastin-like and silk-like hydrogels—bevacizumab could easily be packed into the drug delivery system for extended release. From their testing, 80% of the bevacizumab was released from the competent ECE hydrogel in 20 days; the encapsulated bevacizumab was released through an initial diffusion-controlled phase (0–11 days) and then a combined hydrogel erosion and diffusion-controlled second phase (12–20 days). Additionally, the ECE hydrogel did not show in vitro cytotoxicity on human retinal pigment epithelial (RPE) cells. The hydrogel also preserved the histomorphology and electrophysiology of the rabbit neuroretina after 2 months of intravitreal injection [111]. The ECE hydrogel shows great potential for the treatment of neovascular AMD, and further testing can help determine its efficacy. 

As a possible application for individuals with diabetic retinopathy or neovascular AMD, Nguyen et al. developed and tested an injectable peptide-based hydrogel for in vivo delivery and inhibition of aberrant neovascularization [112]. By attaching an anti-angiogenic peptide sequence (PRKLYDY) to a fibrillizing domain (K-SLSLSLSLSLSL-K) using a glycine spacer, the researchers hypothesized that the new hybrid peptide can self-assemble into a nanofibrous hydrogel while retaining its anti-angiogenic functionality. This hypothesis was accepted after circular dichroism (CD) spectroscopy, for the solution-phase hydrogel, and Fourier transform infrared (FTIR) spectroscopy, for the dried hydrogel form, confirmed a β-sheet secondary conformation. The retention of anti-angiogenic functionality was confirmed by performing a tube formation assay on human umbilical vein endothelial cells (HUVECs), which demonstrated the anti-angiogenic efficacy and specificity of the hybrid peptide. The researchers note that the hydrogel can be easily syringe aspirated, injected, and reassembled in situ for extended ocular drug delivery. Overall, the study was successful in demonstrating the capabilities of the peptide-based hydrogel for diseases like diabetic retinopathy, and it joins other peptide-based hydrogel studies that have broad ocular applications [113]. The study also suggests that the peptide-based hydrogel can potentially treat other diseases, like glaucoma [114]. 

### 4.2. Liposomes

Liposomes are phospholipid vesicles containing a polar core and a lipophilic bilayer, enabling the delivery of both hydrophilic and lipophilic drugs in a way in which they can be protected from the outside environment [115]. Many properties determine the stability of liposomes in vitro and in vivo, such as charge, lipid composition, surface modification using polymers and ligands, and the number of lamellae; this stability is ultimately important for sustained drug delivery [116]. Liposomal formulations are known to have issues with stability, drug entrapment efficiency, short half-lives, and sterilization [117].

Latanoprost (Xalatan, Pfizer), a lipophilic prostaglandin analog drug that is typically delivered as an emulsion, has been found to be very effective in reducing intraocular pressure for patients with glaucoma but can experience higher penetration resistance (and lower bioavailability) when turned into its hydrolyzed active product, latanoprost acid [118,119]. To overcome this barrier, Natarajan et al. fabricated latanoprost-loaded egg–phosphatidylcholine (EggPC) liposomes (which had a high loading efficiency of 94% ± 5%) using a thin-film hydration technique for sustained delivery after only one subconjunctival injection [120]. Overall, 95% of the latanoprost was associated with the bilayer of the EggPC liposomes, and the remaining 5% existed in the liposome as a free drug. Results showed that the EggPC liposomes reduced intraocular pressure in rabbit eyes for up to 90 days; the intraocular pressure lowering effect was found to be significantly greater than once-daily topical latanoprost. The researchers also achieved a slow and sustained release of 60% of latanoprost in 14 days. These initial reports and findings are promising, and these researchers are now aiming to track the movement of liposomes in ocular structures post injection in addition to tracking the stability of the latanoprost within the liposomes to better understand drug release [120]. Clinical trials using liposomes to encapsulate ocular drugs such as latanoprost are still lacking. However, a Phase 2 clinical trial using Subconjunctival Liposomal Latanoprost (POLAT-001) was completed in 2016 for patients with open-angle glaucoma; however, there are no reports on the results of this trial (NCT02466399). 

In 2018, triamcinolone acetonide (TA)-loaded liposomes were developed as a topical formulation, and these liposomes have been shown to deliver TA in the vitreous cavity and effectively reach retinal tissue [121]. TA is a synthetic corticosteroid that was previously investigated in a pilot study as a treatment for cystoid macular edema after cataract surgery when loaded in liposomes [122]. More recently, however, a TA-loaded liposome formulation (TALF) was used as an adjuvant to intravitreal ranibizumab therapy for neovascular AMD. Navarro-Partida et al. hypothesized that this could enhance the efficacy of ranibizumab while minimizing collateral effects through reduced doses [123]. Patients using the TALF as an adjuvant to anti-VEGF intravitreal injections reported an average of 2.5 injection retreatments throughout 12 months, which is much lower than the reported 13 injections per patient throughout 12 months in a previous large clinical trial [124]. One potential concern of this study, however, is that the concentration of ranibizumab used by the TALF was notably lower compared to conventional intravitreal injections. The researchers emphasized that high concentrations are not always synonymous with higher efficacy, as bioavailability must also be considered. 

Liposomes have also been shown to encapsulate bevacizumab in a few studies. For instance, Abrishami et al. used a dehydration method to encapsulate the drug in a liposomal formation with cholesterol to increase its half-life [125]. After intravitreal injections of the bevacizumab-encapsulated liposomes in rabbit eyes, high drug tolerance was reported through 42 days, and clearance of the drug in the vitreous from the liposome was slower than with the soluble form. The bevacizumab concentration in the vitreous after injection was concluded to be a sufficient concentration of the drug for more than 6 weeks for the treatment of ocular diseases like diabetic retinopathy and potentially other ocular diseases that may benefit from bevacizumab. Another group of researchers also reported promising findings using liposomes to encapsulate bevacizumab. Davis et al. used a novel liposomal formulation using the anionic phospholipid-binding protein annexin A5 [126]. With this novel liposomal formulation, a significant increase in the concentration of the encapsulated bevacizumab was found in the posterior segment of rat eyes and rat retinas in vivo after topical application. 

### 4.3. Nanomicelles

**Figure 3:**
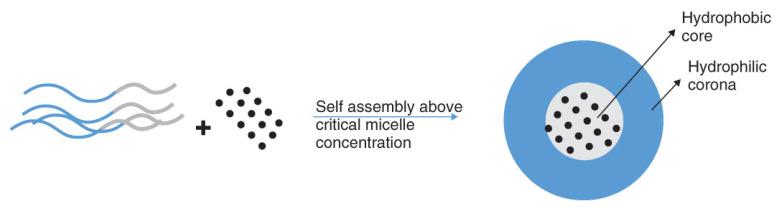
Self-assembly of nanomicelles and drug encapsulation. Reproduced from Vaishya et al. [128] with permission.

Unlike liposomes, which have a hydrophilic head facing the center and external environment, nanomicelles are amphiphilic molecules with hydrophilic head groups that only face the external environment and hydrophobic tails that face the center [127]. Because of this hydrophobic core, this nanocarrier can be used to improve the water solubility of hydrophobic drugs such as dexamethasone. While nanomicelles can be formed into various shapes and sizes based on the molecular weight of their core and corona-forming blocks, self-assembly can occur only above the critical micelle concentration (Figure 3) [128]. It is important to note that the type of nanomicelle carrier, broadly classified as either a spolymeric, surfactant, or polyionic complex, depends on the physicochemical properties of the drug of interest. 

Since dexamethasone is a hydrophobic drug, many researchers have formulated nanomicelles to enhance its solubility in the eye and increase its half-life [129,130,131]. A more recent use of nanomicelles encapsulating dexamethasone is shown by Xu et al., where chitosan oligosaccharide-valylvaline-stearic acid (CSO-VV-SA) nanomicelles were designed for topical ocular drug delivery. This nanomicelle was also based on active targeting of peptide transporter-1 (PepT-1) for enhanced drug permeability and is the first known application of PepT-1-mediated transport in the ophthalmic fields [132]. In their study, the CSO-VV-SA nanomicelles showed sustained release and enhanced penetration through ocular tissues. The researchers also compared their nanomicelle to the recently FDA-approved topical formulation named Cequa—a hydrogenated castor oil-40/octoxynol-40 (HCO-40/OC-40) mixed nanomicelle [133]—which is being used to treat dry eye disease. Cequa has previously been used as a clear, aqueous nanomicellar solution of cyclosporine-A, which is a cyclic non-ribosomal peptide that prevents the activation of T-cells [134]. It has been used to treat a number of diseases, including ocular diseases which involve inflammatory pathways. For comparison purposes, Zu et al. formulated Cequa with dexamethasone. Zu et al. reported that their nanomicelle formulation did not produce any significant cytotoxicity in human corneal epithelial cells and human conjunctival epithelial cells when using their novel nanomicelles and the Cequa nanomicelles. In addition, their novel nanomicelle was comparable with respect to drug loading and delivery efficiency. It was then concluded that this nanomicelle formulation could serve as a possible treatment for macular edema in addition to related ocular diseases like AMD and diabetic retinopathy. 

In other studies, nanomicelles have also been used for the possible treatment of glaucoma. For instance, Ribeiro et al. evaluated single and mixed polymeric nanomicelles of branched poly(ethylene oxide)-poly(propylene oxide) (PEO–PPO) block copolymers of the poloxamine family (Tetronic) [135]. These nanomicelles carried ethoxzolamide, a hydrophobic carbonic anhydrase inhibitor with favorable corneal permeability [136]. Four different poloxamine variants were tested (T908, T904, T1107, T1307), which were chosen based on their hydrophobic blocks being of similar molecular weight and hydrophilic/hydrophobic balance. All variants were hydrophilic and had a similar number of propylene oxide units, though they differed in terms of ethylene oxide content. Among the different variants tested, the T904 variant showed the greatest drug solubilization ability (50-fold increase in apparent solubility), followed by T1107, T1307, and T908. The T904 variant has a lower hydrophilic–lipophilic balance compared to the other variants, which the researchers explained to be an important factor in the solubilization of a drug. Mixed nanomicelles (T904/T1107 and T904/T1307) maintained nearly 100% drug solubility and showed favorable physical stability, which was reported to be greater compared to nanomicelles of only one variant. The researchers also observed sustained release in the 1–5 days range. 

Another study also formulated a nanomicelle for the treatment of glaucoma. Li et al. used nimodipine—a hydrophobic calcium channel blocker that has previously shown benefits in the treatment of glaucoma—for a mixed nanomicelle composed of rebaudioside A (RA) and d-α-tocopheryl polyethylene glycol 1000 succinate (TPGS) [137]. As discussed by the researchers, RA has exhibited great encapsulation of hydrophobic drugs, and TPGS can increase the hydrophobicity of a micelle core if mixed with other amphipathic materials. The mixed nanomicelle formulation aimed to enhance nimodipine’s aqueous solubility for ocular applications. The in vivo pharmacodynamic evaluation suggests that the mixed nanomicelle showed significant intraocular pressure reduction and was well tolerated in rabbit eyes. When compared to commercial timolol maleate ophthalmic drops, the nanomicelle reduced intraocular pressure by more than 10% and the duration of the reduction was approximately twice as long. These findings are promising, and the mixed nanomicelle formulation can be considered for the encapsulation of other hydrophobic drugs in the future for ocular applications. 

### 4.4. DNA-Inspired Nanoparticles

Nanoparticles have been previously used in clinical studies to circumvent problems with surgically implanted capsules through the creation of a drug delivery platform where the drug of interest can reach the back of the eye through a topical solution. For instance, a Phase 2 clinical trial completed in 2019 used cyclodextrin nanoparticles that dissolve in tear fluid to form water-soluble dexamethasone/cyclodextrin complex nanoparticles for the treatment of diabetic macular edema [72]. Stefansson et al. concluded that the nanoparticles were effective in improving central macular thickness, and a Phase2/3 trial that seeks to confirm the findings in a larger population is ongoing. DNA-inspired nanoparticles have also gained attention after many promising results and offer notable advantages in regard to drug delivery [138]. One example is Janus Base Nanotubes (JBNTs), which are novel and biomimetic and self-assemble into long bundles that have hollow channels for drug encapsulation [139,140]. JBNTs include rosette nanotubes (RNTs) which are composed of guanine and cytosine DNA base pairs and six-amino fused adenine and thymine DNA base pairs (AATs) that exhibit excellent biocompatibility and biodegradability [141]. Outside of drug delivery, JBNTs have also been used to increase cell functions, improve implants, and mimic biomolecules [142,143,144,145]. 

A significant advantage of RNTs, as discussed by Song et al., is their ability to incorporate hydrophobic drugs into their tubular structure through hydrophobic interactions. RNTs can also encourage prolonged drug release in a physiological environment due to their hydrophilic outer surface [139]. A variety of hydrophobic drugs have been discussed in this review, including ethoxzolamide, which in theory can be carried by RNTs for the treatment of glaucoma. Although this has not been directly tested, RNTs were shown to slowly release dexamethasone in a study that also tested if the nanoparticle retained the bioactivity of the drug in osteoblasts. Chen et al. showed that the RNTs were capable of releasing dexamethasone for up to 6 days in a physiological environment and also promoted osteoblast functions [146]. We believe that RNTs may have a possible application in the treatment of ocular diseases like AMD and diabetic retinopathy. 

**Figure 4:**
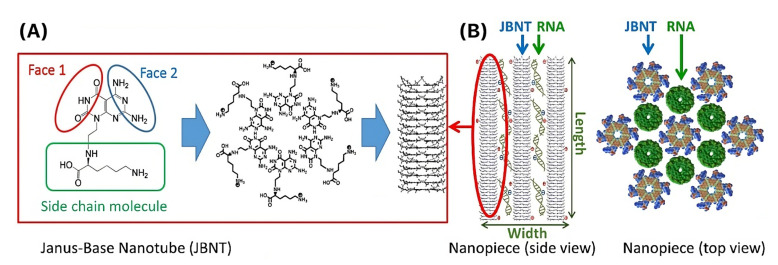
Structure and assembly of (**A**) JBNTs and (**B**) nanopieces with RNA as nano-rod delivery vehicles. Reproduced from Sands et al. [149] with permission.

Small interfering RNA (siRNA) has a wide range of applications, such as treatment of cartilage diseases [147], and was briefly mentioned in Section 2.1. For example, Sirna-027, an siRNA targeting vascular endothelial growth factor receptor-1, can be used for the treatment of neovascular AMD [148]. A few studies have investigated the delivery of RNA using JBNTs, which may have important applications in the treatment of ocular diseases. For instance, Sands et al. developed JBNTs to assemble with small RNAs to form nano-rod delivery vehicles called nanopieces, which were able to deliver the RNA and penetrate through dense extracellular matrices [149]. We believe that this may be applicable to drug delivery that involves large molecules, which have shown poor penetration across the cornea [19]. Figure 4 shows the structure and assembly of the JBNTs and RNA-loaded nanopieces as nano-rod delivery vehicles. 

Additionally, the nanopieces have recently been shown to exhibit better endosome escape than lipid nanoparticles, which is a favorable characteristic considering the challenge of endosomal entrapment of delivered siRNA. In their study, Lee et al. found the nanopieces to be internalized by cells through micropinocytosis, later escaping endosomes via a proton sponge effect that is similar to cationic polymers [150]. The endosomal escape results in this study are very promising and shed light on the capabilities of the JBNT nanoparticles. Other studies have also demonstrated notable uses for JBNTs, such as stem cell adhesion and migration [151,152] and scaffold formation for homeostatic tissue constructs [153]. 

### 4.5. Dendrimers

**Figure 5:**
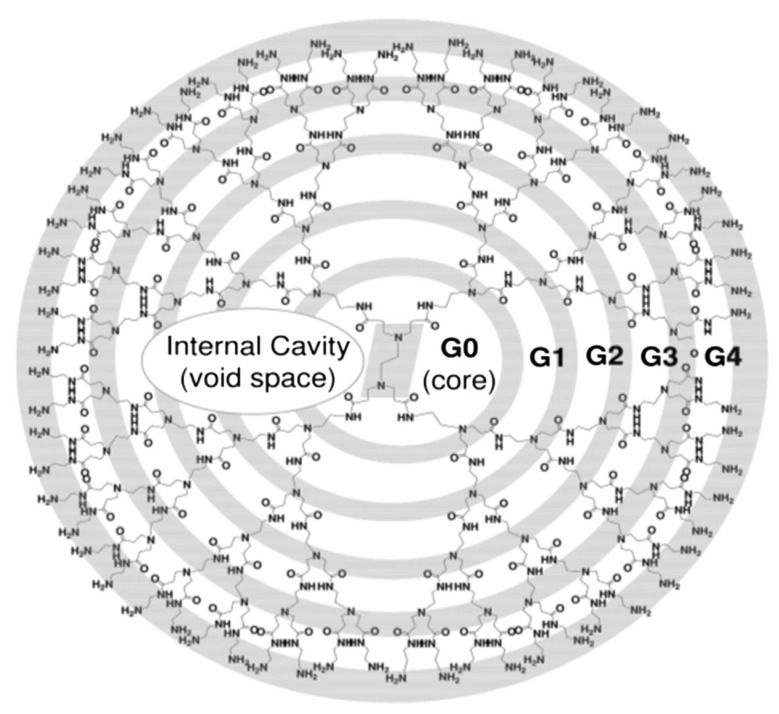
Illustration of a generation 4 (G4) dendrimer with 64 amino groups at the periphery. Image reproduced from Abbasi et al. [154] under the Creative Commons Attribution 4.0 International License (CC BY 4.0).

Dendrimers are branch-like structures formed from repeating growth reactions starting from a multifunctional core. As the diameter increases with each dendrimer generation, the structure begins to adopt a more spherical shape, making it ideal for drug delivery (Figure 5) [154]. Researchers have developed several different types of dendrimers based on their structure to behave like nanomicelles, such as those with a hydrophobic core and a hydrophilic periphery, as well as others with drugs used as surface groups or polymers for increased stability and solubility. However, certain dendrimers have high costs and severe toxicity concerns due to their biodistribution, which researchers are actively attempting to address through new formulations [155]. Lancina et al. in 2018 demonstrated a novel use of dendrimers for ocular drug delivery in their development of DenTimol, a dendrimeric timolol analog for glaucoma therapy [156]. The researchers used polyamidoamine (PAMAM), the most widely studied dendrimer, and conjugated it with the timolol precursor (S)-4-[4-(oxiranylmethoxy)-1,2,5-thiadiazol-3-yl]morpholine (OTM) for the outermost layer and used PEG as a spacer. This design retains the effective structure of timolol and shows no signs of toxicity or irritation. One-time topical treatment of DenTimol on adult Brown Norway male rats showed a 30% reduction in IOP compared to untreated eyes in less than 30 min. The researchers also concluded better drug permeation across the cornea due to DenTimol’s hydrophilic–lipophilic balance (8% vs 3.5% for timolol in 4 h). More testing is needed with other animal models and a complete pharmacokinetic profile is now an area of focus for these researchers. 

Alshammari et al. also used PAMAM dendrimers to develop a therapy involving Ruboxistaurin (RBX), a newly potent investigational anti-VEGF drug for the treatment of diabetic retinopathy [157]. The researchers used PANAM generation 5 dendrimers to encapsulate RBX within its interval cavities, and these dendrimers were characterized across different RBX:PANAM complexes (1:1, 2.1:1, and 5:1). The 5:1 RBX:PANAM complexes showed the greatest loading capacity and highest drug release and did not affect cell viability in macroglial Müller cells. In vivo studies are still needed to evaluate the permeability properties of the dendrimers. 

Dexamethasone has also been incorporated into dendrimers as a possible therapy for AMG and glaucoma. Yavuz et al. combined solutions of generation 3.5 or 4.5 PAMAM into dexamethasone to create a DEX-PAMAM conjugate, where dexamethasone resided on the dendrimer’s surface [158]. Drug release studies revealed an extended duration of dexamethasone release and very slow enzymatic degradation of the DEX-PAMAM conjugates when using cornea and sclera–choroid–retinal pigment epithelium tissues isolated from New Zealand albino rabbit eyes. Intravitreal injection of fluorescent-labeled formulations on male Sprague Dawley rats showed a clearance time of about 24 h and increased the amount of dexamethasone that reached the retina following subconjunctival application. The researchers also indicated that the dendrimers traveled to the retina before clearing the eyes. 

## 5. Microneedles

Microneedles (MNs), also referred to as microneedle arrays or microarray patches, are microscopic injections designed to penetrate tissues and deliver a compound of interest. Typically, several microscopic injections are held together by a single side of a supporting base, and each has a height of less than 2000 µm [159]. Earlier applications of MNs included intradermal drug delivery and have now inspired researchers to explore MN-based drug delivery systems for ocular tissues such as the cornea and sclera.

**Figure 6:**
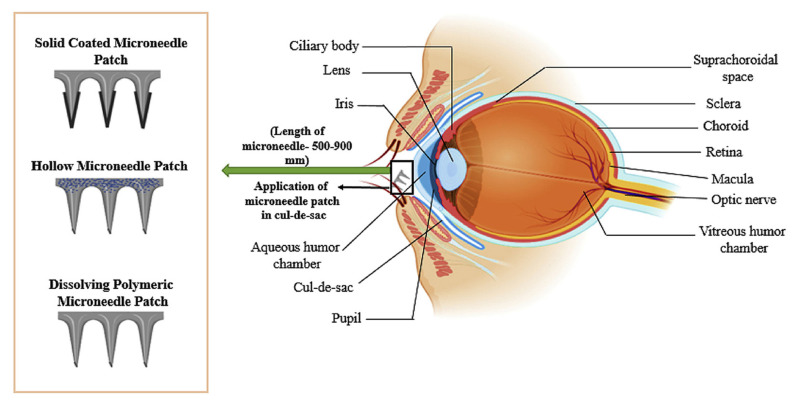
Three main types of microneedle patches for ocular drug delivery. Reproduced from Gupta et al. [160] with permission.

For ocular drug delivery, three types of MNs are most commonly used: solid-coated MNs, hollow MNs, and dissolvable MNs (Figure 6). All three can be used for MN patches, whereas hollow MNs can also be supported by a hypodermic needle. Once placed, solid-coated MNs create micron-sized channels on ocular tissue, which allows for the coating formulation on the surface of the MN to dissolve immediately. Hollow MNs instead only have formulations inside the needles and may make use of nanoparticles to carry the compound of interest. Once contacting the ocular tissue, the formulation is released and the MN patch can be removed. Dissolvable MNs are typically developed using biodegradable polymers and address some of the disadvantages of solid-coated and hollow MNs, such as fabrication and applicability [160]. MNs can also be distinguished from one another based on a number of other characteristics, including but not limited to their geometry, the materials used, the number of needles per patch, their ability to dissolve, and their separability from the supporting base. 

An early application of MNs for the treatment of AMD was presented by C. Lee et al. in 2015 for the use of the Tower Microneedle (TM), a hollow MN supported by a hypodermic needle that can be used to intravitreally inject anti-VEGF antibodies to the posterior segment of mouse eyes [161]. An appealing characteristic of the TM is its significantly thinner needle profile. With an outer needle diameter that is twice as small as the 30-gauge hypodermic needle, the TM is ideal for avoiding the surgical complications that are observed with typical intravitreal injections. In the study, the researchers compared the delivery efficiency for anti-VEGF antibodies between the TM and a 30-gauge hypodermic needle and also compared bleb formation through sodium fluorescein injections (subconjunctival reflux). Angiographic images showed similar delivery efficiency between both groups and lower blebbing for the TM, proving that the TM is a suitable replacement for intravitreal injections. More recently, Jung et al. used a hollow MN to inject a newly designed in situ-forming hydrogel containing bevacizumab and hyaluronic acid crosslinked with poly(ethylene glycol) diacrylate. The researchers used the 750 μm long MN to inject the hydrogel formulation in the suprachoroidal space (SCS), the area between the sclera and choroid that provides direct access to choroidal neovascularization but which causes rapid drug clearance. Crosslinking bevacizumab with hyaluronic acid increased viscosity and reduced drug diffusivity, ultimately achieving slow release from the hydrogel. Bevacizumab retention time was reported to be 6 months from the in vivo rabbit tests; however, more pharmacokinetic studies are needed to verify if these levels are therapeutic.

As a possible application for glaucoma, YeJin Lee et al. developed a rapidly detachable MN pen (RD-MNP) containing a porous dissolvable sacrificial layer with the intended use of inserting it into the sclera [162]. The RD-MNP is made of three main components: the base, a porous water-soluble layer made of poly(vinyl alcohol) and poly(vinyl pyrrolidone), and the drug-loaded tip. Tests using a porcine eye showed almost immediate detachment and embedment of the drug-loaded tip from the MN upon insertion into the sclera using a spring-loaded applicator. This is attributed to the porous structure of the water-soluble layer, which yielded favorable dissolution rates for test drugs compared to a solid structure due to the fast absorption of water molecules. With an instant detachment time and sustained release of ocular drugs, the RD-MNP demonstrates a desirable use of MNs for glaucoma and other prominent ocular diseases. 

**Figure 7:**
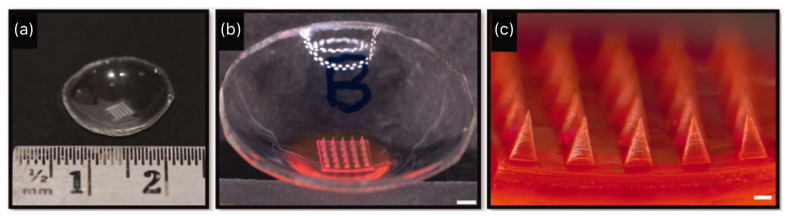
Digital photograph of a dissolvable polymeric microneedle patch supported by a convex polymer (**a**), stereomicroscopic image with a scale bar of 1 mm (**b**), and a magnified stereomicroscopic image with scale bar of 200 µm (**c**). Figure 7 was reproduced from Roy et al. [164] with permission.

While there are currently no ongoing clinical trials for MN arrays/patches, several researchers have demonstrated their effectiveness through in vitro studies. In 2007, Jiang et al. showed for the first time that solid-coated MNs can be used to deliver drugs via intrascleral and intracorneal routes [163]. In their study, the MNs were fabricated from stainless steel sheets using an infrared laser to lengths between 500 and 750 μm. Using the “dip-coating” method, the MNs were coated with an aqueous solution containing pilocarpine hydrochloride, a drug that has been widely used for the treatment of glaucoma. In vitro insertion tests on non-preserved human cadaveric sclera showed the drug coating rapidly dissolving into the scleral tissue within 30 s of insertion. More recently, Roy et al. successfully fabricated a dissolvable polymeric MN patch made of polyvinyl alcohol (PVA) and polyvinyl pyrrolidone (PVP) to carry pilocarpine hydrochloride [164]. To prepare the MN patch, the researchers used the micro molding technique (Figure 7). A mixture of pilocarpine, PVA, and PVP containing a 1 mg equivalent of pilocarpine was spiked into 25 pyramidal micro-cavities (552  ±  1.64 µm length and 255  ±  2.23 µm base width) in a convex-shaped mold mimicking a contact lens. Subsequently, a pilocarpine-free mixture of PVA and PVP was added to the rest of the mold. The in vitro release study demonstrated that 100% of the pilocarpine was released within 30 min of MN patch placement in PBS, and 90% permeated across cornea the in 6 h. Additionally, the ex vivo study using a porcine eye showed greater pilocarpine availability in the aqueous humor within 30 min of applying the MN patch compared to a pilocarpine solution formulation. 

## 6. Drug-Loaded Contact Lenses

**Figure 8:**
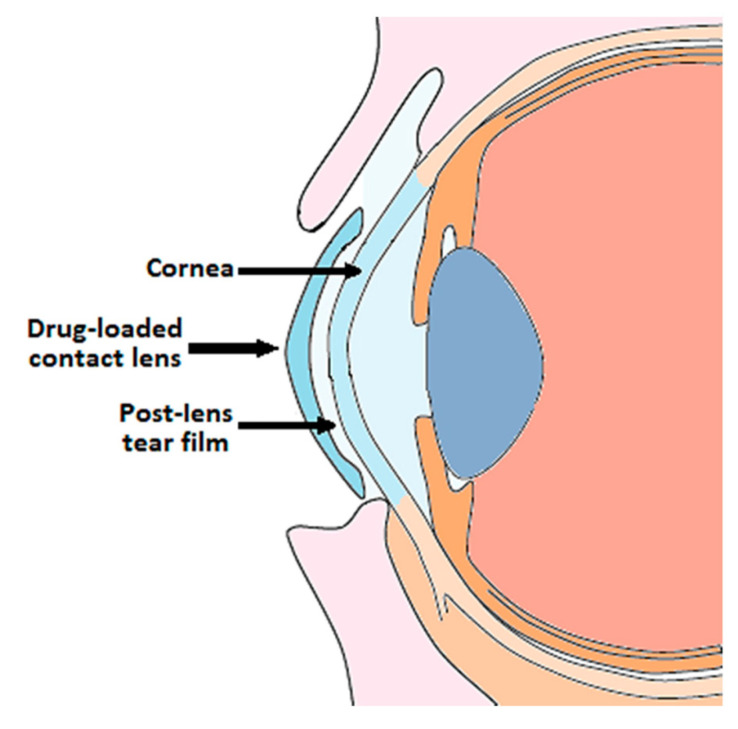
Placement of a drug-loaded contact lens on the cornea for ocular drug delivery. Image reproduced from Franco et al. [166] under the Creative Commons Attribution 4.0 International License (CC BY 4.0).

Contact lenses that have been loaded with ophthalmic drugs have been a growing area of interest due to greater corneal bioavailability and extended drug release compared to eye drops. Once a contact lens is placed on the tear film, the tear film splits into a pre-lens tear film (PLTF) and post-lens tear film (POLTF), with the contact lens able to deposit the drug of interest into both [165]. Drugs released into the PLTF can be absorbed into the conjunctiva or enter the systemic circulation via the canaliculi, whereas drugs released into the POLTF can diffuse directly into the cornea (Figure 8). Recently, researchers are aiming to develop contact lenses that have controllable release durations longer than a few hours or days to reduce frequent lens replacement. To develop such lenses, some have incorporated nanocarriers in their design. At the time of this publication, no clinical trials using drug-loaded contact lenses were found to treat the diseases discussed in this manuscript. However, this section will highlight notable examples of contact lenses used for the treatment of ocular diseases and will introduce the work of researchers who incorporated nanocarriers to address certain limitations of traditional drug-loaded contact lenses. 

A conventional yet simple method for carrying the drug of interest within the contact lens and depositing it into the ocular tissue is the “soak and release” approach, in which a commercial, unmodified lens is soaked in a drug solution and is then placed on the cornea. After C.C. Peng et al. demonstrated that commercial silicone contact lenses loaded with vitamin E can act as a diffusion barrier for hydrophilic drugs to increase the release duration to 1 day, the researchers later published an updated study using vitamin E and timolol maleate to demonstrate a pharmacodynamically favorable extended release duration of 4 days for the treatment of glaucoma [167,168]. In their study, ACUVUE^®^ TruEye™ (Narafilcon A) silicone hydrogel contact lenses, manufactured by Johnson & Johnson Vision Care, were loaded with timolol maleate and vitamin E for in vivo studies with beagle dogs. Results show that intraocular pressure was reduced significantly by continuously wearing the timolol-loaded contact lenses with 20% vitamin E loading. 

While C.C. Peng and coworkers had promising results, the duration of soaking for vitamin E-loaded lenses was increased to 21 days (compared to 7 for control lenses without vitamin E) due to changes in the mechanical properties of the lenses resulting from the diffusion barriers. Dang et al. recently published a study that highlighted this limitation and other issues from using the soak and release method and revealed a novel application of PEGylated solid lipid nanoparticles for ocular drug delivery using contact lenses [169]. In their study, Dang et al. compared conventional soaked contact lenses with latanoprost-loaded PEGylated solid lipid nanoparticles (LP-pSLNs). The conventional lenses were prepared by soaking casted contact lenses in a simulated tear fluid (STF) containing latanoprost for 10 days, whereas the LP-pSLN contact lenses were soaked with STF containing the LP-pSLNs for 10 days. The in vitro drug release studies show significantly lower burst release, high latanoprost uptake, and sustained latanoprost release for up to 96 h in contact lenses with LP-pSLNs. No symptoms of irritation were observed in a rabbit model, and the researchers believe that the LP-pSLN contact lenses can be a suitable substitute for eye drops in the treatment of glaucoma. 

Recently, a Phase 1 clinical trial began recruiting participants with glaucoma and ocular hypertension for testing of a latanoprost-eluting contact lens (NCT04500574). This trial is based on a pre-clinical study of the contact lenses used on the glaucomatous eyes of cynomolgus monkeys in 2016. In their study, Ciolino et al. assessed the ability of low-dose and high-dose contact lenses to lower intraocular pressure. Results showed a statistically significant reduction in intraocular pressure for both doses compared to the untreated baseline, and these doses were also shown to be as effective as using latanoprost eye drops daily [170]. 

## 7. Discussion

The use of biomaterials in drug delivery systems has enhanced the efficacy of therapeutics in different medical applications. The complex anatomy and protective physiological systems of the human eye that have been huge barriers to effective drug delivery have benefitted from the emergence of biomaterials in ophthalmic drug delivery treatments. The selection of biomaterials for ophthalmic drug delivery is no longer limited to biocompatibility and biodegradability properties. Surface properties, drug loading capability, patient compliance, and convenience are just a few of the many other factors being considered when selecting the proper biomaterial for ocular drug delivery. Therefore, the development of a new, effective biomaterial-based treatment for ocular diseases requires a multifaceted approach that considers all these different factors as the field moves forward.

Prolonged exposure of the eye to the drug alongside the enhanced permeation and targeted delivery of drugs are some of the most significant advantages of the biomaterials that have been developed for ocular disease treatment [17,171,172,173]. Progression of the biomaterial-based ophthalmic drug delivery systems has also been followed by the involvement of other technologies, such as 3D printing, nanotechnologies and functionalized nanoparticles, and microneedling techniques [174,175]. These cutting-edge technologies have enabled the production of more complex and sophisticated drug delivery systems, further augmenting the potential benefits of biomaterials.

Despite the benefits of biomaterials in ocular drug delivery systems, there are still varied challenges limiting treatment efficiency. Table 3 shows the advantages and limitations of the different biomaterials discussed in this review for ocular drug delivery. One of the major considerations is maintaining drug stability and release kinetics, which can be affected by factors such as pH, temperature, and enzymatic degradation in the ocular environment. Additionally, there is a need to improve targeting efficiency to ensure that drugs are delivered to the specific ocular tissue of interest, highlighting the need for careful selection and optimization of biomaterials. The development of effective biomaterial-based drug delivery systems requires a multidisciplinary approach that draws on a range of different technologies and expertise. The utilization of developed technologies combined with the best selection of biomaterials is considered to be the most successful solution in overcoming the mentioned limitations in the treatment of ocular diseases. 

The overall trajectory of drug delivery systems for the management of ocular diseases suggests promising results that can guide the future direction of the field. The continuing progress in nanotechnologies, injectable implants, microneedles, and drug-eluting contact lenses, alongside the advent of gene and stem cell-based therapies, holds considerable potential for the treatment of ocular diseases. We are also noticing a growing number of researchers dedicated to using natural, bioinspired materials for ocular drug delivery. For instance, silk-like hydrogels were previously discussed for the treatment of glaucoma. Ongoing research has shown that silk may also be used to construct porous scaffolds, films, and nanofibers to increase drug retention time [176]. Developments in this area hint that the next generation of biomaterials for ocular drug delivery will be renewable and more eco-friendly. Moreover, there is still a preference for developing biodegradable materials over non-biodegradable materials due to greater biocompatibility and reduced toxicity. With continued research and development, the full potential of biomaterials in ocular drug delivery can be explored, ultimately benefiting patients and advancing medical science.

## 8. Conclusions

In this review, we first introduced some of the most prominent ocular diseases, particularly those affecting the posterior segment, such as AMD, glaucoma, diabetic retinopathy, and diabetic macular edema, and described their conventional treatments. After highlighting some of their limitations, we discussed and provided examples of major classes of drug delivery for their treatment, including non-biodegradable and biodegradable implants, nanocarriers (hydrogels, liposomes, nanomicelles, DNA-inspired nanoparticles, and dendrimers), microneedles, and drug-loaded contact lenses. Significant progress has been made to address the limitations of conventional treatments, such as requiring frequent intravitreal injections. As more drug delivery systems based on biomaterials are developed in the future, we may see more favorable outcomes that enhance patient experience and gain wide acceptance. Besides the FDA-approved therapies discussed in this review, we can expect more in the future as researchers conduct larger studies. 

## Figures and Tables

**Table 1:** Pharmacokinetic properties of some of the most common anti-VEGF drugs.

Drug	Binding Targets	Dose	IV Half-Life (Days)	Serum Half-Life	References
Pegaptanib	VEGF165	0.3 mg (0.9 mL)	3.9 (monkey)	10 days	[52]
Ranibizumab	VEGF-A	0.5 mg (0.05 mL)	2.6–2.88 (rabbit) 3–3.2 (monkeys) 7.1 (human)	0.25 days	
Bevacizumab	VEGF-A	1.25 mg (0.05 mL)	4.32–6.61 (rabbit) 3.1 (monkey) 6.7–10 (human)	21 days	
Aflibercept	VEGF-A, VEGF-B, PGF	2 mg (0.05 mL)	4.5–4.7 (rabbit)	18 days	
Anecortave acetate		15 mg	3–5 (mouse)		[53]
VEGF-Trap	VEGF-A, VEGFR1, VEGFR2, human PlGF, and VEGF-B	0.3 mg/0.03 mL	3.63 days (rabbit)	11.4 days	[35,36,38]

**Table 2:** Notable and ongoing clinical trials using biomaterials for age-related macular degeneration (AMD), diabetic macular edema (DME), diabetic retinopathy (DR), and glaucoma (GL).

Ocular Disease	Biomaterial Type	Clinical Trial ID	Study Phase and Status	Sponsor	Participants	Key Findings or Other Information
DME	Non-biodegradable Implant and Inserts	NCT00344968	Phase 3–completed (2010)	Alimera Sciences	956	An intravitreal insert of fluocinolone acetonide was used for the treatment of diabetic macular edema. Both low- and high-dose FA inserts significantly improved best-corrected visual acuity (BCVA) in participants with DME over 2 years [61].
		NCT04469595	Phase 4–recruiting (expected to be completed in 2024)	Alimera Sciences	300	This clinical trial will compare two treatment regimens for participants with early DME: a fluocinolone acetonide intravitreal implant (ILUVIEN) with supplemental aflibercept versus aflibercept anti-VEGF intravitreal injections.
		NCT04503551	Phase 3–active, not recruiting (expected to be completed in 2024)	Hoffmann-La Roche	174	This clinical trial will evaluate the efficacy, safety, and pharmacokinetics of a Port Delivery System prefilled with 100 mg/mL ranibizumab (PDS) relative to the comparator arm for participants with DR without center-involved DME.
	Biodegradable Implants	NCT02471651	Phase 4–completed (2018)	California Retina Consultants	40	This study was designed to measure the effectiveness of dexamethasone implants versus monthly intravitreal anti-VEGF injections for participants with persistent DME. No reports are available about the results of the clinical trial.
		NCT01492400	Phase 2–completed (2014)	Allergan	363	This clinical trial was designed to compare BCVA between dexamethasone implants (0.7 mg every 5 months) and ranibizumab (0.5 mg). Both were well tolerated by participants with DME and improved BCVA [70].
		NCT01892163	Phase 3–completed (2014)	Moorfields Eye Hospital NHS Foundation Trust	100	This study was designed to compare the efficacy of fixed versus pro re nata dosing of a 700 μg dexamethasone implant in participants with refractory DME. No reports are available about the results of the clinical trial.
		NCT04576689	Phase 2–active, not recruiting (expected to be completed in 2024)	Ripple Therapeutics Pty Ltd.	60	This clinical trial will evaluate the safety and efficacy of the IBE-814 intravitreal implant in participants with DME. The non-polymeric implant is designed to deliver a low, sustained dose of DEX for up to 6 months.
DR	Biodegradable Implant	NCT05695417	Phase 1–recruiting (expected to be completed by 2024)	Ocular Therapeutix, Inc.	21	This clinical trial was designed to evaluate the safety, tolerability, and efficacy of OTX-TKI, a polyethylene glycol-based hydrogel implant for intravitreal use, in participants with moderately severe to severe non-proliferative DR.
DME	Dendrimers	NCT05387837	Phase 2–recruiting (expected to be completed by 2023)	Ashvattha Therapeutics, Inc.	30	This clinical trial will evaluate the safety, tolerability, and pharmacokinetics of a dendrimer (D-4517.2) after subcutaneous administration for participants with AMD or DME. A Phase 1 trial was previously completed in 2022 (NCT05105607).
	Microneedles	NCT03126786	Phase 2–completed (2018)	Clearside Biomedical, Inc.	71	This clinical trial was designed to evaluate the safety and efficacy of a suprachoroidally injected triamcinolone acetonide formulation (CLS-TA) used with intravitreal aflibercept in participants with DME. Results show that this treatment provided similar visual benefits after 24 weeks compared to aflibercept monotherapy [71].
	Nanoparticles	NCT05343156	Phase 2–Completed (2019)	Oculis	144	This clinical trial was designed to evaluate the efficacy and safety of dexamethasone/cyclodextrin nanoparticle eye drops for participants with DME. Results indicate significant improvement in increasing central macular thickness compared to vehicle. Phase 2/3 is ongoing to confirm results in a larger population [72].
AMD	Non-biodegradable Implants	NCT03677934	Phase 3–completed (2021)	Hoffmann-La Roche	415	This clinical trial was designed to assess the efficacy, safety, and pharmacokinetics of ranibizumab delivered via the Port Delivery System (PDS) compared with ranibizumab intravitreal injections at 0.5 mg (10 mg/mL) in participants with AMD. While participants preferred PDS over intravitreal injections, both provided high participant satisfaction [73].
	Biodegradable Implants	NCT02087085	Phase 2–terminated	Allergan	310	This clinical trial was designed to assess the safety and efficacy of brimonidine intravitreal implants in participants with geographic atrophy due to AMD. The implant showed favorable safety outcomes and reduced lesion growth at month 12. Phase 3 development is supported [74].
		NCT03630315	Phase 1–active, not recruiting (expected to be completed in 2022)	Ocular Therapeutix, Inc.	29	This clinical trial was designed to evaluate the safety, tolerability, and efficacy of OTX-TKI, a polyethylene glycol-based hydrogel implant for intravitreal use, in participants with AMD. Study results have not been posted.
GL	Non-biodegradable Implants	NCT03519386	Phase 3–active, not recruiting (expected to be completed in 2024)	Glaukos Corporation	1000	This clinical trial is designed to compare the safety and efficacy of iDose^®^ TR, a travoprost-containing intraocular implant, and a timolol maleate ophthalmic solution (timolol) for participants with open-angle glaucoma or ocular hypertension. The trial will investigate two different elution rates from the intraocular implant against timolol.
	Biodegradable Implants	NCT05822245	Phase 1 and 2–recruiting (expected to be completed in 2026)	Perfuse Therapeutics, Inc	36	This clinical trial is designed to investigate the ocular and systemic safety and tolerability of the PER-001 bioerodible intravitreal implant. Researchers will be examining the sustained release of PER-001, a small molecule endothelin receptor antagonist, to selectively target inhibition of the endothelin pathway. Endothelin is known as a potent vasoconstrictor and regulator of vascular tone.
		NCT01157364	Phase 1 and 2–completed (2016)	Allergan	109	This clinical trial was designed to evaluate the safety and efficacy of the biodegradable bimatoprost sustained-release implant (Bimatoprost SR) for participants with open-angle glaucoma or ocular hypertension. Results show rapid and sustained reductions in intraocular pressure over 6 months and a favorable efficacy and safety profile over 24 months [75].
		NCT04060758	Phase 1–recruiting (expected to be completed in 2023)	PolyActiva Pty Ltd.	40	This clinical trial is designed to identify a safe and officious dose of latanoprost-free acid using the Prezia biodegradable implant, a cross-linked polytriazole hydrogel. The researchers aim to use the hydrogel implant for participants with open-angle glaucoma.
	Liposomes	NCT02466399	Phase 2–completed (2016)	Peregrine Ophthalmic	80	This clinical trial was designed to compare Subconjunctival Liposomal Latanoprost (POLAT-001), a liposomal formulation of latanoprost, to latanoprost solution for participants with open-angle glaucoma and ocular hypertension. No reports are available about the results of the clinical trial.
	Contact Lenses	NCT04500574	Phase 1–recruiting (expected to be completed in 2023)	Massachusetts Eye and Ear Infirmary	31	This clinical trial is designed to assess the safety, tolerability, comfort, and feasibility of a latanoprost-eluting contact lens. Participants will either use the latanoprost-eluting contact lens or a commercial contact lens with a regiment of latanoprost eye drops.

**Table 3:** Advantages and limitations of different biomaterials for ocular drug delivery.

Biomaterial Type	Advantages	Limitations
Non-biodegradable implants	The structure of the implant does not change upon implantation. It can be transplanted directly into the vitreous body. The structure of the implant is highly modifiable before implantation. Several materials can be used to achieve the desired drug release rate. The implant can be made small enough for ocular use through the use of microelectromechanical technology.	Can cause complications similar to intravitreal injections due to the requirement of a surgical procedure. An additional surgical procedure is required to remove the implant if the implant malfunctions, if complications such as discomfort arise, or if the drug of interest needs to be replenished.
Biodegradable implants	The Implant can be broken down into smaller nontoxic fragments, which can be absorbed or excreted by the body. There is limited impact on eye health. Only requires a single surgical procedure. Implant can take the form of many shapes, such as plugs, pellets, disks, and sheets.	Hydrolysis of the biodegradable polymer may lead to a “final burst stage” after reaching its critical point, causing uncontrollable release of the remaining drug within the implant.
Hydrogels	Hydrogels are able to swell while retaining their structure to carry the drug of interest. Can be used as an implant or as particles for injectable use. High biocompatibility and ability to hold and release drugs makes it a favorable biomaterial. Several hydrogel structures can be fabricated according to the type of polymer (natural, synthetic, or hybrid), its durability, and its composition (homopolymer, copolymer, or semi-interpenetrating network).	Clinical studies are lacking and further testing is needed to measure safety and efficacy in participants with ocular diseases. Synthetically derived hydrogels may break down into toxic by-products. Sterilization of hydrogels can alter their structure and release profile.
Liposomes	Polar core and lipophilic bilayer allow for the delivery of both hydrophilic and lipophilic drugs. They are made of natural materials and can easily be synthesized in a laboratory. Can be used to protect bioactive agents from the surrounding environment.	The stability of the liposome must be controlled in order to achieve a stable release profile. Liposomal formulations are known to have issues with stability, drug entrapment efficiency, short half-lives, and sterilization.
Nanomicelles	Hydrophobic core can be used to improve the water solubility of hydrophobic drugs such as dexamethasone. Self-assembly of nanomicelles can occur if it is above critical micelle concentration. Can be formed into various shapes and sizes based on the molecular weight of the core and corona-forming blocks.	Drug retention time remains an issue when using nanomicelles due to eye clearance mechanisms. Some nanomicelle formulations can be used topically, but others may require injections that could cause discomfort for patients.
DNA-inspired nanoparticles	DNA-inspired nanoparticles are biomimetic and can self-assemble into a structure that promotes drug encapsulation. They possess excellent biocompatibility and biodegradability. Can be used to incorporate hydrophobic drugs into its structure. Hydrophilic outer surface encourages prolonged drug release.	Research and development of DNA-inspired nanoparticles is newer than most other biomaterials and will require further testing to prove its safety and efficacy among patients with ocular diseases.
Dendrimers	Dendrimers can adopt a spherical structure after a sufficient amount of branching from the core, making them ideal for ocular drug delivery. Can be fabricated to behave like nanomicelles by having a hydrophobic core and a hydrophilic periphery. Polymers can be used as surface groups to increase stability and solubility.	A complete pharmacokinetic profile and testing with a variety of animal models are still needed. Permeability properties still need to be investigated. Higher generation dendrimers are costly and severely toxic. Intravitreal injections may still be needed, causing discomfort for patients.
Microneedles	Can be used with nanoparticles to encapsulate and release a drug of interest. Dissolvable microneedles are developed with biodegradable polymers, allowing for safer use. Microneedle patches can be easily inserted on the surface of the eye.	No clinical trials exist to date that test the safety and efficacy of microneedles among patients with ocular diseases. The amount of drug to be delivered is limited.
Drug-loaded contact lenses	Has greater corneal bioavailability and extended drug release compared to commercial eye drops. Can be incorporated with other biomaterials in the design. The application of contact lenses is safe and easy for most individuals.	Lack of a mechanism to control the release of the drug of interest. Drug release durations are still mostly between a few hours to a day and will require frequent lens replacement. Clinical studies are lacking and further testing is needed to measure safety and efficacy among participants with ocular diseases.

## Data Availability

No new data were created in this study. Data sharing is not applicable to this article.
